# Development of Mouse-Tumor Model Using Prostate Cancer (PC3) Cell Line for High-Intensity Focused Ultrasound (HIFU) Ablation

**DOI:** 10.1155/proc/5678314

**Published:** 2025-09-06

**Authors:** Nabin Khanal, Victoria Summey, Jeffrey Bailey, Xin Duan, Yi Zheng, Liang Zhu, Keith Stringer, Marepalli Rao, Rupak K. Banerjee

**Affiliations:** ^1^Department of Mechanical and Materials Engineering, University of Cincinnati, Cincinnati, Ohio, USA; ^2^The Comprehensive Rodent and Radiation Shared Facility, Cincinnati Children's Hospital Medical Center, Cincinnati, Ohio, USA; ^3^Experimental Hematology and Cancer Biology, Cincinnati Children's Hospital Medical Center, Cincinnati, Ohio, USA; ^4^Department of Mechanical Engineering, University of Maryland Baltimore County (UMBC), Baltimore, Maryland, USA; ^5^Division of Pathology and Laboratory Medicine, Cincinnati Children's Hospital Medical Center, Cincinnati, Ohio, USA; ^6^Environmental and Public Health Sciences, University of Cincinnati, Cincinnati, Ohio, USA; ^7^Department of Biomedical Engineering, University of Cincinnati, Cincinnati, Ohio, USA

**Keywords:** HIFU, mouse-tumor model, noninvasive, PC3, prostate cancer, ultrasound

## Abstract

High-intensity focused ultrasound (HIFU) is a noninvasive modality that is gaining prominence for the localized treatment of malignant tumors. Most current HIFU research utilizes mouse tumor models, where selection of appropriate mouse breed is important for conducting thermal ablation experiments on tumors with consistency. In this study, three breeds (NOD/SCID GammaC −/− (NSG), NSG-SGM3 (NSGS), and Homozygote J:NU (Nude); *n* = 2 per group) originating from Jackson Laboratory were tested for identifying the breed that has sufficient size for conducting HIFU experiments. Tumors were developed using a human PC3 (CRL-1435) prostate cancer cell line and monitored over 5 to 7 weeks. The surface area and volume of the implanted tumors were determined by assuming the tumor having an ellipsoidal shape. At the end of the growth period, NSG mice exhibited 29% larger tumor surface area than NSGS and 58% larger than Nude mice. Similarly, NSG mice had a 55% larger tumor volume than NSGS mice and 100% larger than Nude mice. Therefore, this research established NSG mice as the superior mouse breed for the PC3 cell–induced tumor growth having established size within a reasonable timeline (5–7 weeks). Subsequently, the NSG model with a larger sample size (*n* = 48) was selected for HIFU ablation, and histopathological analysis revealed a significantly higher number of apoptotic cells in the HIFU-treated tumors compared to controls. This further confirmed the model's suitability for HIFU research. Tumor surface area and volume compared between the tested (*n* = 2) and selected (*n* = 48) groups were statistically insignificant (*p* = 0.78 for surface area and *p* = 0.60 for volume).

## 1. Introduction

High-intensity focused ultrasound (HIFU) stands out as a noninvasive modality gaining prominence for the localized heating treatment of deep-seated malignant tumors. To enhance the effectiveness of the thermal therapy during the HIFU procedures, researchers have developed diverse in vitro [1–3] and in vivo [4–6] models to test the efficacy of HIFU. The literature reveals a spectrum of experiments involving in vitro models employing tissue-mimicking materials (TMMs) and turkey/chicken breasts, and in vivo models featuring mice. Currently, a significant amount of HIFU research revolves around utilizing mouse tumor models to evaluate the thermal dosage needed for damaging tumor cells.

In the area of cancer research, an array of mouse strains is readily available, primarily sourced from Jackson Laboratory in Bar Harbor, ME. The judicious selection of a mouse strain is important for conducting seamless experiments in tumor studies. Comprehensive Rodent and Radiation Shared Facility at Cincinnati Children's Hospital and Medical Center (CCHMC) locally houses 3 mouse strains, all originating from Jackson Laboratory: NOD/SCID GammaC−/− (NSG [7, 8]—#005557), NSG-SGM3 (NSGS [9–12]—#013062), and Homozygote J: NU (Nude [13, 14]-#007850). These three types of mice were assessed in this study to determine a suitable mouse tumor model for the HIFU studies.

The NSG mice, with their severe immunodeficiency due to SCID and IL2rgnull mutations, have become pivotal in cancer research. Their compromised immune system allows efficient engraftment of human cells, facilitating studies on tumor biology and tests of potential cancer therapies. The NSGS mice, an advancement in cancer research models, express human IL3, GM-CSF, and SCF. This modification enhances the engraftment of myeloid lineages and regulatory T cells, making them particularly relevant for immuno-oncology studies. These mice provide a superior platform for investigating complex interactions between the immune system and cancer. Nude mice, characterized by athymia and immunodeficiency, have been fundamental in cancer research for decades. Their lack of T cells enables successful transplants of tumor cell xenografts, contributing to studies on tumor growth and drug testing in oncology. While not capturing full immune complexity, Nude mice remain relevant in specific contexts, offering insights into tumor biology and early-stage drug evaluations. A study by Jiang et al. [15] reported the use of nude mouse models for HIFU procedures involving the treatment of pancreatic cancer.

A human PC3 (CRL-1435) prostate cancer cell line used for developing the tumor model in mice is provided by American Type Culture Collection (ATCC, Manassas, Virginia). The PC3 cell line is initiated from a bone metastasis of grade IV prostatic adenocarcinoma from a 62-year-old, White, male. The PC3 prostate cancer cell line was selected for HIFU studies due to its androgen-independent, highly metastatic nature, which closely models the advanced, treatment-resistant prostate cancer [16]. Its high tumorigenic and invasive potential makes it especially relevant for evaluating ablation therapies aimed at aggressive tumors [17]. PC3 cells also exhibit resilience under stress conditions such as hypoxia and thermal insult, mimicking the tumor microenvironment (TME) during HIFU exposure [18]. Furthermore, PC3 cell line has been used in ultrasound-based therapies [18–20] for the treatment of prostate cancer. Additionally, PC3 cell line was used previously in a study [21] for the quantification of thermal dose using micro-CT Hounsfield unit. For these reasons, the PC3 cell was considered an appropriate choice for establishing a mouse tumor model for HIFU ablation therapies.

Tsingotjidou et al. [22] created a mouse model using PC3 cells to study the metastasis of prostate cancer in adult human bone. When the mice developed tumors, they were sacrificed to harvest tumor fragments. One tumor fragment from single PC3 neolacZ-injected animal was implanted in five SCID mice. They observed that 3 mice had tumors spread to skeletal system, 1 mouse showed tumor around human bone implant, 2 mice showed liver and kidney metastasis, and 1 mouse showed lung metastasis. McGovern et al. [23] studied humanization of PCa model in NSG mice to observe the effect of primary TME on metastasis. They observed that humanization did not affect primary tumor size, but reduced bone metastasis, increased liver metastasis, and did not change lung and spleen metastasis. This suggests that humanization influences site-specific PCa spread. Koshida et al. [24] compared the two in vivo models for prostate cancer, orthotopic and intratesticular inoculation of LNCaP and PC3 cells. They observed PC3 to be highly tumorigenic and metastatic in both models. Although mouse tumor models developed in the above-mentioned studies provide significant understanding of prostate cancer, these are not designed for HIFU ablation applications.

Several in vivo studies have been performed using different strains of mice for HIFU ablation therapies. For instance, Beik et al. [25] used male Balb/c mice, Devarakonda et al. [4] used C57BL/6J mice, and Larrat et al. [26] used rats (no strains mentioned). This shows a lack of established mouse models for HIFU research to ensure consistency for comparing the results from different studies. The significance of the current study is establishing a robust mouse tumor model based on tumor size and growth and validating the model with immunohistochemistry by observing its response after HIFU ablation. This will reduce the time and resources required to identify an appropriate mouse model in future studies, allowing researchers to focus on optimizing cancer treatment parameters using HIFU.

To the best of our knowledge, an established mouse tumor model for HIFU studies has not been previously reported. Therefore, this study aims to determine an appropriate mouse model for conducting HIFU research to achieve fast growth of the PC3 tumors within a time duration of several weeks.

## 2. Methods

### 2.1. Experimental Procedure

The in vivo experimental cohort was comprised of two male mice from each of the three locally available strains, totaling six mice. All animal studies were approved by the Institutional Animal Care and Use Committee (IACUC2021-0042) at CCHMC (Cincinnati, OH, US). To establish the cell line–derived xenograft model, 6 to 8-week-old male mice with a bodyweight around 30 g were engrafted subcutaneously with 1 × 10^7^ PC3 cells into the right flank. Mice were group-housed under pathogen-free and temperature- and humidity-controlled environmental conditions (21 ± 1.5 °C temperature, 55 ± 10% humidity, and a 12-h light–dark cycle).

### 2.2. Cell Culture and Administration

PC3 cells were obtained from ATCC and cultured in F-12K medium supplemented with 10% fetal bovine serum (FBS) and 1% penicillin–streptomycin. The medium was filtered multiple times to ensure sterility. Upon thawing, cells were centrifuged, counted, and seeded into 75-cm^2^ tissue culture flasks, then incubated at 37°C until reaching confluence (typically within 2-3 days). Subculturing was performed using 0.25% Trypsin-0.53 mM EDTA for detachment, followed by reseeding at a 1:3 split ratio. All procedures were conducted under aseptic conditions, with ethanol-sterilized equipment. The culture medium was renewed 2–3 times per week, maintaining consistency across subsequent passages.

For the injection procedure, PBS served as the medium, and a 0.2-mL solution containing the requisite cell count was injected using a 27-gauge needle. Postinjection, all mice underwent observation for abnormal behavior. Tumor monitoring involved regular measurements 2 to 3 times weekly over a 5–7-week growth period. The area surrounding the tumor was consistently shaved for NSG and NSGS to facilitate clear observation. Shaving was not needed for Nude mice as they were devoid of hair. The tumor measurement was conducted using digital calipers.

### 2.3. Scheduling

Initially, six mice (two from each of three tested strains) were used to evaluate tumor growth characteristics. Based on this assessment, one strain was selected for further experimentation. All six mice from the tested group, along with 48 additional mice of the selected strain, underwent HIFU ablation to evaluate the feasibility of applying focused ultrasound in this tumor model. Since the HIFU procedure requires approximately 40–45 min per mouse, an efficient scheduling strategy was implemented. The six mice from the tested group were treated on the same day, while the 48 mice from the selected group were divided into six groups of 7–10 animals and treated in a staggered manner. This grouping and scheduling approach ensured consistent HIFU application following tumor growth evaluation across both study groups.

### 2.4. Tumor Size Calculations

The tumor was assumed to be an ellipsoid, and the following formula [27] was used to calculate the surface area of an ellipsoid.

(1) $$\begin{array}{c} \text{SA}={\left(\frac{4\pi \left({\left(ab\right)}^{1.6}+{\left(ac\right)}^{1.6}+{\left(bc\right)}^{1.6}\right)}{3}\right)}^{1/1.6}. \end{array}$$

The following formula [28] was used to calculate the volume of an ellipsoid:

(2) $$\begin{array}{c} V=\frac{4}{3}\pi abc, \end{array}$$

where *a*, *b*, and *c* are the three dimensions of the ellipsoid.

Identical methodologies were adopted for both tested and selected groups as discussed in the next section.

### 2.5. Statistical Analysis

Functional data analysis using spline models [29–31], such as B-splines, is particularly well suited for small sample size studies, as it allows for the extraction of meaningful trends and derivatives (e.g., velocity and acceleration) from longitudinal data. By smoothing over time, spline models reduce the impact of individual measurement fluctuations while preserving the overall shape and dynamics of the data. This leads them to be ideal for analyzing tumor growth trajectories when only a limited number of animals are available per group. For this reason, the spline model was fitted for tested mouse groups. The mouse breed was selected based on the velocity and acceleration of tumor growth. To assess the statistical significance of the findings, the area under the curve (AUC) [32, 33] was calculated using 10,000 simulated paired observations across three groups: NSG, NSGS, and Nude. For each simulation [32, 33], the group means of AUC were computed, and the proportion of instances in which the group with the highest tumor growth exceeded the AUCs of the other two groups was determined.

For the selected mouse group having higher sample size (*n* = 48), linear regression models were applied to evaluate tumor growth dynamics. The line of best fit was computed using the least squares method. To determine statistical differences in tumor growth between the tested group and selected group, Student's *t*-test was conducted. A *p* value of less than 0.05 was considered statistically significant. The tumor volume and surface area are presented as mean ± SE (standard error). All statistical analyses were performed using R-markdown (RStudio Team (2022), Boston, MA) software.

### 2.6. Magnetic Resonance–HIFU (MR-HIFU) System

A clinical MR-HIFU system (Sonalleve V2, Philips Medical Systems, Vantaa, Finland), integrated into a 1.5 T whole-body scanner (Philips Ingenia, Healthcare, Best, The Netherlands) was used for scanning and sonicating the mouse tumors. The MR-HIFU system has a 256-element phased array HIFU transducer that was used to focus energy on small volumes within the tumor. The diameter of the transducer was 140 mm, and the operating frequency was 1.2 MHz. More details about the MR-HIFU system can be found in our previous work [4]. For sonication of tumor, the mice were placed above the HIFU transducer on the MR-HIFU table.

### 2.7. Tissue Processing and Staining for Histopathology

Four hours after HIFU application, mice were euthanized using carbon dioxide followed by the secondary physical method of cervical dislocation (approved by IACUC2021-0042). Tumors were immediately harvested, photographed, and fixed in neutral buffered formalin prior to paraffin embedding. Four consecutive sections with a thickness of 4 μm (16 μm in total) were cut at multiple sampling sites, the sites located 330 μm apart from one another. For histopathologic analysis, the first tumor section was stained with H&E, while the second section was stained with cleaved caspase 3 (CC3) antibody using diaminobenzidine as chromogen for immunohistochemistry.

Quantitative analysis of CC3 antibody–stained tissue sections was performed using the positive pixel count software Leica (Leica Microsystems Inc., Buffalo Grove, IL) on scanned images. The apoptotic index was calculated as the ratio (Nsr) of the number of strongly positive cells (Nsp) to the total number of cells (Nt) as shown below.

(3) $$\begin{array}{c} \text{Nsr}=\frac{\text{Nsp}}{\text{Nt}}. \end{array}$$

A higher Nsr value indicates a greater proportion of apoptotic cells. For each tissue section, rectangular regions of identical dimensions were defined using the software to systematically capture the entire section. The three regions with the highest Nsr values were selected, and their average was used for comparative analysis across samples.

## 3. Results

Growth of tumors using the surface area and volume measurements over time is reported in this section. Growth of PC-3 tumor cells is compared among the selected three strains of mice. Data collection commenced after the 10th day postinjection of tumor cells as the initial size of tumors are relatively small.

### 3.1. Testing of Mouse Model

**Figure 1:**
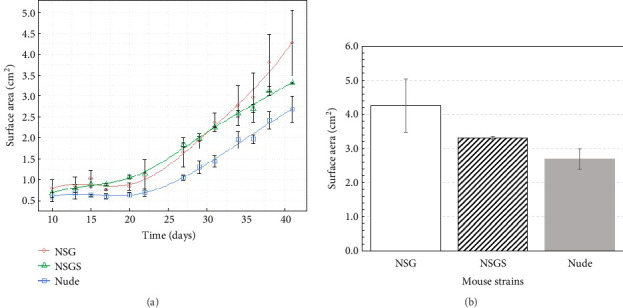
(a) Tumor surface area (cm^2^) growth over time for different strains of mouse and (b) tumor surface area (cm^2^) on last day.

**Figure 2:**
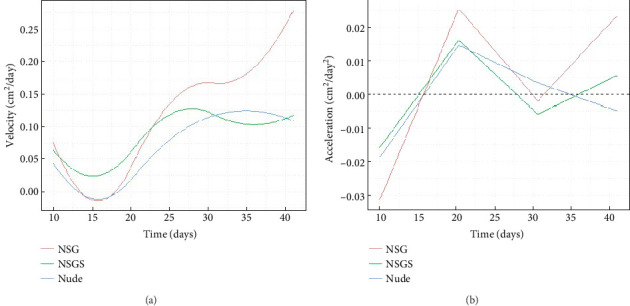
(a) Velocity of tumor growth (cm^2^/day) and (b) acceleration of tumor growth (cm^2^/day^2^) over time for different strains of mice.

**Figure 3:**
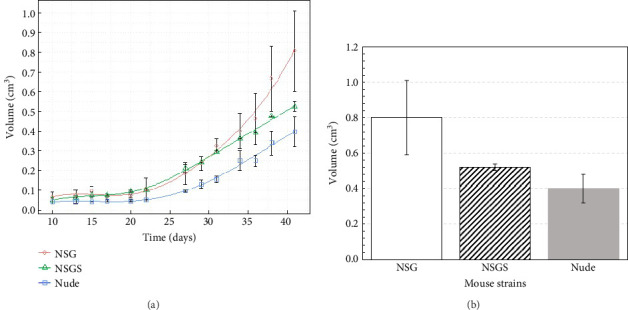
(a) Tumor volume (cm^3^) growth over time for different strains of mouse and (b) tumor volume (cm^3^) on last day.

**Figure 4:**
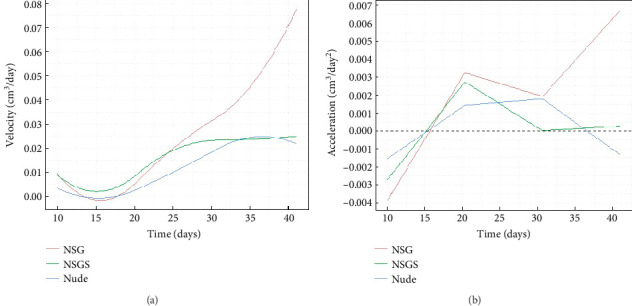
(a) Velocity of tumor growth (cm^3^/day) and (b) acceleration of tumor growth (cm^3^/day^2^) over time for different strains of mice.

To investigate tumor growth dynamics in three mouse strains (NSG, NSGS, and Nude), tumor surface area and volume data are analyzed over a period from Day 10 to Day 41 postinoculation. Functional data analysis is employed to fit B-spline [29–31] curves (nbasis = 6) to the combined tumor surface and volume data for each strain. The results are presented in Figures 1 and 2, which depict the curves (trend lines) for surface area, velocity, and acceleration, respectively, with standard errors and raw data points included for surface area. Similar results are obtained for volume in Figures 3 and 4.

#### 3.1.1. Tumor Surface Area

Figure 1(a) illustrates the temporal progression of smoothed tumor surface area (cm^2^) for different strains (NSG, NSGS, and Nude) of mouse using spline model [29–31]. Error bars represent standard errors of the mean surface area, calculated from two mice per strain, indicating variability within each group. Data points (open symbols: circle for NSG, triangle for NSGS, square for Nude) mark the observed mean surface areas at each measurement time point. NSG mice exhibited the highest tumor growth, reaching 4.26 ± 0.79 cm^2^ by Day 41, followed by NSGS at 3.31 ± 0.01 cm^2^ and Nude at 2.69 ± 0.30 cm^2^, as shown in Figure 1(b). This indicates that NSG mice have a tumor surface area that is 29% larger than NSGS mice and 58% larger than Nude mice by Day 41. The NSG strain consistently showed larger tumor surface areas compared to NSGS and Nude mice after Day 30.

Figure 2(a) illustrates the velocity of tumor growth (cm^2^/day) across the three strains. NSG mouse displayed the highest peak velocity (∼0.26 cm^2^/day around Day 41), indicating a rapid increase in tumor size during the later stages. NSGS and Nude mice showed lower peak velocities (∼0.12 cm^2^/day around Day 26 and ∼0.12 cm^2^/day around Day 35, respectively). All strains exhibited near-zero or somewhat negative velocities early, suggesting a slow initial (Days 10–15) growth phase, followed by a steady increase in velocity after Day 20.

Figure 2(b) presents the acceleration of tumor growth (cm^2^/day^2^) for the three strains. Acceleration profiles reveal dynamic changes in growth rates. NSG mouse showed the highest positive acceleration (∼0.02 cm^2^/day^2^ around Days 20 and 41). NSGS and Nude mice exhibited lower peak accelerations (∼0.01 cm^2^/day^2^ around Day 20 for both) compared to NSG mice. Negative acceleration values for all breeds suggest an initial (Days 10–15) deceleration before growth rates increase.

#### 3.1.2. Tumor Volume

Figure 3(a) illustrates the temporal progression of smoothed tumor volume (cm^3^) for different strains (NSG, NSGS, and Nude) of mouse using spline model [29–31]. Error bars represent standard errors of the mean volume, calculated from two mice per strain, indicating variability within each group. Data points (open symbols: circle for NSG, triangle for NSGS, square for Nude) mark the observed mean volumes at each measurement time point. NSG mouse exhibited the highest tumor growth, reaching 0.80 ± 0.21 cm^3^ by Day 41, followed by NSGS at 0.51 ± 0.02 cm^3^ and Nude at 0.40 ± 0.08 cm^3^ as shown in Figure 3(b). This indicates that NSG mice have a tumor volume that is 54% larger than NSGS mice and 100% larger than Nude mice by Day 41. The NSG strain consistently showed larger tumor volumes compared to NSGS and Nude after Day 30.

Figure 4(a) illustrates the velocity of tumor growth (cm^3^/day) across the three strains. NSG mouse displayed the highest peak velocity (∼0.07 cm^3^/day around Day 41), indicating a rapid increase in tumor size during the later stages. NSGS and Nude mice showed lower peak velocities (∼0.02 cm^3^/day for both around Day 35). All strains exhibited near-zero or somewhat negative velocities, suggesting a slow initial (Days 10–15) growth phase, followed by a steady increase in velocity after Day 25.

Figure 4(b) presents the acceleration of tumor growth (cm^3^/day^2^) for the three strains. NSG mouse showed the highest positive acceleration (∼0.0035 cm^3^/day^2^ around Day 20 and ∼0.007 cm^3^/day^2^ around Day 41). NSGS and Nude mice exhibited lower peak accelerations (∼0.0025 cm^3^/day^2^ around Day 20 and ∼0.002 cm^3^/day^2^ around Day 30, respectively). Negative acceleration values for all breeds suggest an initial (Days 10–15) deceleration before growth rates increase.

#### 3.1.3. Statistical Significance

Paired simulations [32, 33] comprising 10,000 iterations were performed across the three mouse strains: NSG, NSGS, and Nude, using both surface area and volume data from two mice per group. AUC was calculated over the interval from Day 30 to Day 41, which represents the most relevant phase of tumor growth in this experimental model. In 76% of the simulations using surface area and 83% of the simulations using volume, the NSG strain yielded the highest AUC, indicating a markedly greater tumor growth potential compared to the other strains. These findings provide strong support for selecting the NSG strain as the preferred model for tumor growth studies. This simulation-based approach enabled robust statistical evaluation despite the limited sample size.

### 3.2. Assessment of Selected Mouse Model

After the above selection process, a group of 48 NSG mice are selected for further assessment. The total of 48 mice were divided into six groups. Groups 1 through 4 had 7 mice, whereas groups 5 and 6 had 10 mice. Each group was injected with PC3 tumors on different dates, resulting in staggered timelines for tumor size measurement. The day of injection of PC3 cells for each group was considered to be the starting point of the day count in all figures. This was done to ensure efficient scheduling of experiments. All groups underwent a consistent 5–7 weeks of growth period. Each data point on a particular day of measurement represents the average area or volume of individual groups.

**Figure 5:**
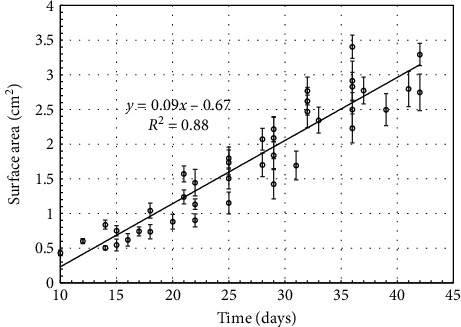
Tumor surface area growth with time for selected NSG group.

Tumor measurement started around the tenth day following PC-3 cells injection considering initial small size of the tumors. Such smaller size is also observed in testing groups.  Figure 5 shows temporal growth of tumor surface area. This figure shows the surface area of the tumor at different timepoints measured till the end date (42 days) of the experiment. A linear fit of the surface area as shown in Figure 5 had a slope of 0.09 and an intercept of 0.68 with an *R*^2^ value of 0.88.

**Figure 6:**
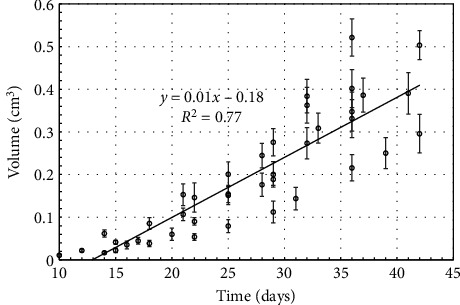
Tumor volume growth with time for selected NSG group.

Figure 6 shows temporal growth of tumor volume in 48 NSG mice of the selected group. A linear regression plot is plotted for the data points. The linear fit as shown in Figure 6 has a slope of 0.014 and *y*-intercept of 0.18 with an *R*^2^ value of 0.78.

**Figure 7:**
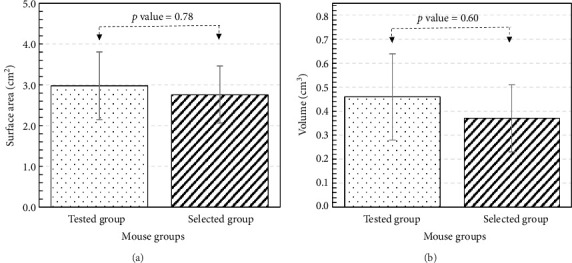
Comparison of surface area and volume between tested NSG group and selected NSG group.

Student's *t*-test is performed to compare the size of the tumor (surface area and volume) between tested NSG group and selected NSG group. The *p* values obtained are 0.78 and 0.60, as shown in Figure 7, indicating the differences in both surface area and volume between the tested NSG group and the selected NSG group are statistically insignificant. Overall, a similar trend between tested and selected groups of NSG mice supports the validity of model selection.

### 3.3. Histopathology Using H&E Staining and Immunohistochemistry

**Figure 8:**
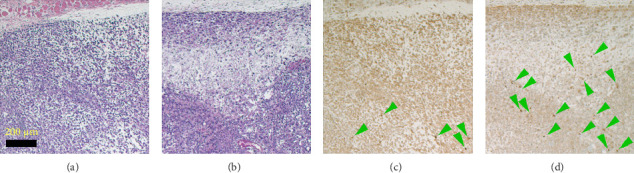
Representative histopathology images of H&E-stained tissue sections of (a) control case: tumor without the application of HIFU energy (0 W) and (b) tumor receiving HIFU energy of 30 W. Corresponding locations of immunohistochemical images of apoptosis marker, CC3, are shown in (c) control case: tumor without the application of HIFU energy (0 W) and (d) tumor receiving HIFU energy of 30 W. Several cells that are rendered dark brown by immunohistochemical detection of the CC3 are marked with green arrowheads. All the images are pictured at 40X magnification, and the scale bar represents 200 microns.

MR scanning followed by HIFU treatment was performed on the NSG mouse model.  Figure 8 shows representative histopathology images of hematoxylin and eosin-stained tissue sections of (Figure 8(a)) control case: tumor without HIFU treatment (0 W) and (Figure 8(b)) tumor receiving HIFU energy of 30 W. Several cells rendered dark brown by immunohistochemical detection of the apoptosis marker, cleaved caspase, in those same regions are highlighted in green arrowheads (Figure 8(c) (0 W) and Figure 8(d) (30 W), respectively). Light microscopic examination of H&E-stained sections of tumor-bearing tissues revealed localized changes in regions receiving HIFU energy. As illustrated in Figure 8, panel “B,” representative of a tumor exposed to HIFU energy, tissue disruption was present in the form of widespread cellular debris and an absence of cohesive intact tumor cell populations. These may represent energy-absorbing areas affected by associated heat and vaporization. Dark brown and condensed fragments of nuclei in and near these regions demonstrate apparent apoptosis (Figure 8, panel “D,” stained using an antibody directed against the noted apoptosis marker). In contrast, in the case of tissues without HIFU energy application, the marked tissue disruption changes are not present (panel “A”) and the frequency of tumor cells demonstrating apparent apoptosis appears lower (panel “C”).

Table 1 presents the quantification of apoptotic area based on the ratio (Nsr) of strongly positive cells (Nsp) to the total number of cells (Nt). In the control tumor section, which did not receive HIFU energy, the Nsr value is low at 0.17. An increase to 0.31 (82% increase) is observed for the tumor section which received HIFU energy.

This analysis demonstrates a clear increase in apoptotic activity in tumors treated with HIFU, validating that the observed tumor tissue is biologically responsive and viable for therapeutic evaluation. The presence of functionally relevant cellular processes, such as apoptosis, strengthens the reliability of the model beyond just tumor size.

## 4. Discussion

The current mouse model is specifically developed for HIFU ablation experiments. Even though the developed mouse model was only used for HIFU treatment, it can also be used for other methods of treatment like proton therapy, X-ray therapy, and gamma ray therapy.

Nude mice are easier to handle as they are devoid of fur compared to NSG and NSGS mice, which need shaving fur in the tumor zone. Despite this advantage, the growth of tumor surface area and volume for Nude mice is extremely low compared to the other two breeds. Further, the presence of host immune TME in Nude mice can be preferable for studies that focus on immune response. However, inferior tumor growth can be a deterrent for studies that focus on physical ablation therapies like HIFU that act directly on tumors. Hence, for HIFU ablation, superior tumor growth is a more valuable quality than better immune response. The superior ability of NSG mice for rapid tumor growth and enhanced tumor engraftment makes them preferable for HIFU ablation studies. Hence Nude mice may not be suitable in comparison to NSG mouse model for conducting HIFU ablation experiments in a reasonably shorter timeline (5–7 weeks). They may be suitable for HIFU ablation experiments with a longer timeline (beyond 7 weeks).

Limited research has been done on developing mouse models using prostate cancer cells. While the mouse models developed by Tsingotjidou et al. [22], McGovern et al. [23], and Koshida et al. [24] have contributed valuable insights into prostate cancer progression and metastasis, these models were not designed with HIFU ablation applications in mind. Their primary focus was on studying tumor behavior, metastasis patterns, and the effects of TME rather than therapeutic interventions. Additionally, existing in vivo HIFU studies have employed various animal strains, such as Balb/c mice [25], C57BL/6J mice [4], and rats [26]. This shows lack of established mouse model specifically for HIFU research. This inconsistency limits the ability to compare therapeutic outcomes across studies. The current study addresses this critical gap by developing a robust and reproducible mouse tumor model tailored for HIFU ablation, supported by tumor growth metrics and immunohistochemical response. This model lays the foundation for more consistent and efficient preclinical research, enabling future studies to focus on optimizing HIFU treatment parameters instead of spending time and resources on selection of mouse models.

The histopathology studies using H&E stains revealed the localized presence of elongated nuclei and nuclear fragmentation in the HIFU-treated tumor sections (Figure 8(b)) which is consistent with previous research [4]. Examination using immunohistochemistry to detect apoptosis events using CC3 antibody demonstrated the presence of confluent signatures of apoptosis in HIFU-treated tumors. Similar signatures have been attributed to apoptosis and thermal ablation in previous works [4, 34]. Furthermore, cleaved caspase-positive cells have been widely characterized as apoptotic cells in the literature [35]. Additionally, green fluorescent protein (GFP) has been used as a cellular marker tool and application of such GFPs needs to be studied in future [36].

While the current study establishes a foundational mouse model for HIFU applications, there are several areas for improvement. Future studies could expand the range of mouse strains by incorporating additional cell lines available from sources such as the Jackson Laboratory, potentially uncovering models with improved tumor growth characteristics or treatment responses. The current work involves small sample size (*n* = 2) per strain. Increasing the number of mice per group would enhance the statistical robustness of the model. Although digital calipers provide a practical method for measuring tumor dimensions, measurement of depth is somewhat challenging. For ablation-relevant intensities/temperatures having cavitation, improved thermal dose models [3] of cell death are needed. In future, alternative methods such as imaging can be utilized for effective tumor size measurement.

## 5. Conclusion

This study establishes the NSG mouse as a suitable preclinical model for prostate cancer research, particularly in the context of HIFU applications. NSG mice exhibited more consistent and substantial tumor growth compared to other strains, resulting in significantly larger tumor volumes and surface areas over the study period, particularly between 30-day and 41-day period. This enhanced tumor development enables improved assessment of therapeutic interventions. Histopathological analysis revealed a higher frequency of CC3 positive cells in HIFU-treated tumors, indicating increased apoptosis and supporting the effectiveness of HIFU in inducing death of tumor cells. The significance of these findings lies in demonstrating the suitability of the NSG model for cancer studies. Moreover, the results reinforce the applicability of the NSG mouse model in preclinical evaluation and optimization of noninvasive, MR imaging–guided tumor ablation therapies with potential clinical impact.

## Figures and Tables

**Table 1:** Quantification of apoptotic cells using the ratio of strongly positive cells to total positive cells.

0 W	30 W
0.17	0.31 (82% increase)

## Data Availability

The data that support the findings of this study are available upon request from the corresponding author. The data are not publicly available due to privacy or ethical restrictions.

## References

[1] Devarakonda S. B., Myers M. R., Lanier M., Dumoulin C., Banerjee R. K. (2017). Assessment of Gold Nanoparticle-Mediated-enhanced Hyperthermia Using MR-Guided High-Intensity Focused Ultrasound Ablation Procedure. *Nano Letters*.

[2] Bera C., Devarakonda S. B., Kumar V., Ganguli A. K., Banerjee R. K. (2017). The Mechanism of nanoparticle-Mediated Enhanced Energy Transfer During High-Intensity Focused Ultrasound Sonication. *Physical Chemistry Chemical Physics*.

[3] Nandlall S. D., Bazán-Peregrino M., Mo S., Coussios C.-C. (2012). Determination of Cytotoxic Thermal Dose During HIFU Ablation. *AIP Conference Proceedings*.

[4] Devarakonda S. B., Stringer K., Rao M., Myers M., Banerjee R. (2019). Assessment of Enhanced Thermal Effect due to Gold Nanoparticles During MR-Guided High-Intensity Focused Ultrasound (HIFU) Procedures Using a Mouse-Tumor Model. *ACS Biomaterials Science & Engineering*.

[5] Khanal N., Marciniak M., Daniel M. C. Functionalized Nanoparticles Mediated High Intensity Focused Ultrasound (HIFU) Ablation in Mice.

[6] Khanal N., Michael M., Daniel M. C. Thermal Effects of Fab-Functionalized Gold Nanoparticles During High Intensity Focused Ultrasound (HIFU) Ablation in Mice.

[7] Shultz L. D., Lyons B. L., Burzenski L. M. (2005). Human Lymphoid and Myeloid Cell Development in NOD/LtSz-scid IL2R Gamma Null Mice Engrafted With Mobilized Human Hemopoietic Stem Cells. *The Journal of Immunology*.

[8] Coughlan A. M., Harmon C., Whelan S. (2016). Myeloid Engraftment in Humanized Mice: Impact of Granulocyte-Colony Stimulating Factor Treatment and Transgenic Mouse Strain. *Stem Cells and Development*.

[9] Billerbeck E., Barry W. T., Mu K., Dorner M., Rice C. M., Ploss A. (2011). Development of Human CD4+FoxP3+ Regulatory T Cells in Human Stem Cell factor-Granulocyte-Macrophage Colony-Stimulating Factor-and Interleukin-3-Expressing NOD-SCID IL2Rγ(Null) Humanized Mice. *Blood*.

[10] Janke L. J., Imai D. M., Tillman H. (2021). Development of Mast Cell and Eosinophil Hyperplasia and HLH/MAS-Like Disease in NSG-SGM3 Mice Receiving Human CD34+ Hematopoietic Stem Cells or Patient-Derived Leukemia Xenografts. *Veterinary Pathology Online*.

[11] Wunderlich M., Chou F. S., Link K. A. (2010). AML Xenograft Efficiency is Significantly Improved in NOD/SCID-IL2RG Mice Constitutively Expressing Human SCF, GM-CSF and IL-3. *Leukemia*.

[12] Bryce P. J., Falahati R., Kenney L. L. (2016). Humanized Mouse Model of Mast Cell-Mediated Passive Cutaneous Anaphylaxis and Passive Systemic Anaphylaxis. *Journal of Allergy and Clinical Immunology*.

[13] Flanagan S. P. (1966). Nude, a New Hairless Gene with Pleiotropic Effects in the Mouse. *Genetical Research*.

[14] Pantelouris E. M. (1968). Absence of Thymus in a Mouse Mutant. *Nature*.

[15] Jiang L., Hu B., Guo Q., Chen L. I. (2012). Treatment of Pancreatic Cancer in a Nude Mouse Model Using High-Intensity Focused Ultrasound. *Experimental and Therapeutic Medicine*.

[16] Kaighn M. E., Narayan K. S., Ohnuki Y., Lechner J. F., Jones L. W. (1979). Establishment and Characterization of a Human Prostatic Carcinoma Cell Line (PC-3). *Investigative Urology*.

[17] Rocchi P., Jugpal P., So A. (2006). Small Interference RNA Targeting heat-shock Protein 27 Inhibits the Growth of Prostatic Cell Lines and Induces Apoptosis via caspase-3 Activation in Vitro. *BJU International*.

[18] Lee H. J., Yoon Y. I., Bae Y. J. (2016). Theragnostic Ultrasound Using Microbubbles in the Treatment of Prostate Cancer. *Ultrasonography*.

[19] Wei C., Bai W. K., Wang Y., Hu B. (2014). Combined Treatment of PC-3 Cells with Ultrasound and Microbubbles Suppresses Invasion and Migration. *Oncology Letters*.

[20] Arora J. S., Murad H. Y., Ashe S. (2016). Ablative Focused Ultrasound Synergistically Enhances Thermally Triggered Chemotherapy for Prostate Cancer in Vitro. *Molecular Pharmaceutics*.

[21] LeBrun A., Joglekar T., Bieberich C., Ma R., Zhu L. (2016). Identification of Infusion Strategy for Achieving Repeatable Nanoparticle Distribution and Quantification of Thermal Dosage Using micro-CT Hounsfield Unit in Magnetic Nanoparticle Hyperthermia. *International Journal of Hyperthermia*.

[22] Tsingotjidou A. S., Ahluwalia R., Zhang X., Conrad H., Emmanouilides C. (2003). A Metastatic Human Prostate Cancer Model Using Intraprostatic Implantation of Tumor Produced by PC-3 neolacZ Transfected Cells. *International Journal of Oncology*.

[23] McGovern J. A., Shafiee A., Wagner F. (2018). Humanization of the Prostate Microenvironment Reduces Homing of PC3 Prostate Cancer Cells to Human Tissue-Engineered Bone. *Cancers (Basel)*.

[24] Koshida K., Konaka H., Imao T., Egawa M., Mizokami A., Namiki M. (2004). Comparison of Two in Vivo Models for Prostate Cancer: Orthotopic and Intratesticular Inoculation of LNCaP or PC-3 Cells. *International Journal of Urology*.

[25] Beik J., Abed Z., Ghadimi-Daresajini A. (2016). Measurements of Nanoparticle-Enhanced Heating from 1MHz Ultrasound in Solution and in Mice Bearing CT26 Colon Tumors. *Journal of Thermal Biology*.

[26] Larrat B., Pernot M., Aubry J. F. (2010). MR-Guided Transcranial Brain HIFU in Small Animal Models. *Physics in Medicine and Biology*.

[27] Poelaert D., Schniewind J., Janssens F. (2011). Surface Area and Curvature of the General Ellipsoid.

[28] Moler C. (1961). Mathematical Handbook for Scientists and Engineers. *Engineering and Science*.

[29] Balakrishnan N., Kannan N., Nagaraja H. N. (2004). Advances in Ranking and Selection, Multiple Comparisons, and Reliability: Methodology and Applications: Springer Science & Business Media.

[30] Gibbons J. D., Olkin I., Sobel M. (1979). An Introduction to Ranking and Selection. *The American Statistician*.

[31] Hong L. J., Fan W., Luo J. (2021). Review on Ranking and Selection: A New Perspective. *Frontiers of Engineering Management*.

[32] Riesthuis P., Otgaar H., Bücken C. (2025). Ready to ROC? A Tutorial on Simulation-Based Power Analyses for Null Hypothesis Significance, Minimum-Effect, and Equivalence Testing for ROC Curve Analyses. *Behavior Research Methods*.

[33] Gu J., Ghosal S., Roy A. (2008). Bayesian Bootstrap Estimation of ROC Curve. *Statistics in Medicine*.

[34] Zhou Y. F. (2011). High Intensity Focused Ultrasound in Clinical Tumor Ablation. *World Journal of Clinical Oncology*.

[35] Dull A. B., Wilsker D., Hollingshead M. (2018). Development of a Quantitative Pharmacodynamic Assay for Apoptosis in Fixed Tumor Tissue and Its Application in Distinguishing Cytotoxic drug-induced DNA Double Strand Breaks from DNA Double Strand Breaks Associated with Apoptosis. *Oncotarget*.

[36] Yanar S., Sarihan M., Kasap M., Akpinar G., Teke K., Yaprak Bayrak B. (2024). GFP Transfection Alters Protein Expression Patterns in Prostate Cancer Cells: A Proteomic Study. *Journal of Fluorescence*.

